# Executive Function Scale (UEF-1) in Cuban University students: dataset

**DOI:** 10.3389/fpsyg.2025.1483879

**Published:** 2025-05-20

**Authors:** Diego D. Díaz-Guerra, Carlos Ramos-Galarza, Marena de la C. Hernández Lugo, Yunier Broche-Pérez

**Affiliations:** ^1^Psychology Department, Universidad Central Marta Abreu de Las Villas, Santa Clara, Villa Clara, Cuba; ^2^Facultad de Salud y Bienestar, Carrera de Psicología Clínica, Pontificia Universidad Católica del Ecuador, Quito, Ecuador; ^3^Prisma Behavioral Center, West Palm Beach, FL, United States

**Keywords:** executive functions, university students, UEF-1, neuropsychological evaluation, self-regulated behavior

## Specifications data

- Subject: Psychology.
- Specific subject area: Developmental and Educational Psychology.
- Data collection: This research was based on a cross-sectional study. An online survey was conducted via Google Forms^®^ from January to March 2024, distributed through WhatsApp groups, Facebook, email lists, and websites. No incentives were offered for participation. All Cuban citizens over 18 years old currently enrolled in university studies in the country were eligible to participate. The Executive Function Scale (UEF-1) for university students, which consists of 31 items and assesses 7 dimensions, was used.
- Data source location: Universidad Central “Marta Abreu” de Las Villas. Villa Clara, Santa Clara, Cuba (22°24'49''N 79°57'58''O).
- Repository name: Mendeley Data.
- Data identification number: doi: 10.17632/j5zjb46tr7.1.
- Direct URL to data: https://data.mendeley.com/datasets/j5zjb46tr7/1.

## Value of the data

- This data provides a detailed perspective on executive functions among Cuban university students and helps fill a gap in the academic literature on this specific topic.
- Collected via an online survey and based on a scale with demonstrated psychometric properties, this data offers a methodological model that can be replicated in other studies of executive functions across different cultural contexts.
- With a substantial sample size of 1,092 university students, the results of this survey provide a solid foundation for future comparisons and analyses in similar populations or other educational settings.
- These data can serve as a reference for future research on executive functions in university environments, enabling other researchers to expand knowledge in this specific area.

## Introduction

In the university setting, students face a variety of challenges, such as organizing resources to complete tasks, planning their learning, monitoring their progress across subjects, and ensuring their work meets required standards (Díaz-Guerra et al., 2025a). Additionally, they must assess whether their learning strategies are effective and maintain metacognitive awareness of their actions to grow into competent professionals (Wang and Yang, 2021; Ramos-Galarza et al., 2020; Cushman et al., 2022).

This study introduces the dataset from the University Executive Function Scale (UEF-1), a measure originally developed and validated for Spanish-speaking populations in Ecuador and Chile (Ramos-Galarza et al., 2023) and later validated in the Cuban context (Díaz-Guerra et al., 2025a). This scale has been used to develop an interactive model for understanding university students' ability to self-verify their learning in Cuban, Chilean, and Ecuadorian populations (Díaz-Guerra et al., 2024), as well as to propose explanatory models of conscious monitoring of responsibilities and self-regulated learning behaviors among Cuban University students (Díaz-Guerra et al., 2025b).

In this context, studying executive functions is particularly important. Executive functions are a system of higher-order cognitive processes that enable behavioral self-regulation, conscious control of thoughts and actions, and flexible adaptation to complex situations to achieve specific goals. These high-level cognitive skills enable students to consciously monitor and regulate their educational development in academic settings (Ramos-Galarza et al., 2023; Cartwright et al., 2019).

This research focuses on seven specific executive functions (Díaz-Guerra et al., 2025a):

(a) Conscious monitoring of responsibilities: Active tracking of obligations to ensure their fulfillment.
(b) Supervisory attention system: Mechanism regulating focus and allocation of attention.
(c) Conscious regulation of behavior: Deliberate control of actions to align with goals and norms.
(d) Verification of learning-related behavior: Assessment of actions that facilitate knowledge acquisition.
(e) Decision-making: Process of choosing between alternatives based on reasoning and objectives.
(f) Conscious regulation of emotions: Intentional management of affective responses to maintain balance.
(g) Management of resources for task completion: Efficient organization of time, effort, and tools to achieve goals.

The dataset presented in this study explores the executive functions of university students in Cuba, providing insights into how these functions operate within a Latin American context.

## Methods

### Procedures

To ensure an effective data collection process, the Executive Function Scale (UEF-1) was administered online using Google Forms. This platform enabled efficient, standardized data collection while offering participants the flexibility to complete the survey at their convenience. Participation was entirely voluntary, with students free to withdraw from the study at any time.

Informed consent was obtained with a focus on transparency, ensuring that participants were fully aware of the study's aims and procedures. Participants were made aware of the scope of their involvement and the implications of sharing their information, ensuring that their participation was based on an informed and voluntary decision.

### Assessment tools

The study employed the following instruments:

*Demographic Information:* Demographic data collected included age, gender, and the province in which the students were enrolled in university.

*University Executive Function Scale (UEF-1):* Developed and validated for Spanish-speaking populations, this scale consists of 31 items that assess seven executive functions relevant to the university setting. These functions are: conscious monitoring of responsibilities, supervisory attention system, conscious regulation of behavior, verification of behavior for learning, decision-making, conscious regulation of emotions, and management of resources for task completion. Responses were measured on a 5-point Likert scale, ranging from 1 (strongly disagree) to 5 (strongly agree).

### Data analysis procedure

The UEF-1 was meticulously selected for its relevance in evaluating executive functions within the university environment. Its structure and content were carefully aligned with the research objectives, facilitating a comprehensive and detailed assessment of participants' executive capabilities. Robust data security measures were implemented to maintain participant confidentiality and anonymity. The dataset does not include personally identifiable information, such as names or addresses, which could reveal participant identities. Additionally, explicit consent was obtained from participants for the anonymous sharing of their sociodemographic data and survey responses. Statistical software JASP was utilized for in-depth analysis and to derive meaningful insights from the dataset. JASP is an open-source, free software that provides a broad array of statistical and graphical tools, enabling a thorough exploration of the data and the identification of significant patterns and relationships. The presented data include participants' sociodemographic characteristics and confirmatory factor analysis to evaluate the scale's psychometric properties. This report provides descriptive statistics, item-test correlations (r), and factor loadings for the UEF-1 items.

### Data analysis

The study included a sample of 1,092 participants from Cuban universities who completed the Executive Function Scale (UEF-1) (Díaz-Guerra et al., 2025a). Data were collected from January 23 to May 3, 2024, using snowball sampling across 14 higher education institutions nationwide, with voluntary participation and no missing data. Sample characteristics are presented through descriptive statistics (Table 1), including age (*M* = 20.05, *SD* = ±1.72 years), gender (60.4% male), and geographic distribution (93.0% Central Region). Reliability estimates were high for the total score (α = 0.91, ω = 0.91), while factor analysis revealed predominantly medium values for conscious monitoring of responsibilities (α = 0.76, ω = 0.77), supervisory attention system (α = 0.83, ω = 0.83), behavior verification for learning (α = 0.76, ω = 0.76), decision-making (α = 0.70, ω = 0.70), conscious regulation of emotions (α = 0.82, ω = 0.82), and task-solving element management (α = 0.76, ω = 0.76), with medium-low values for conscious regulation of behavior (α = 0.65, ω = 0.65).

**Table 1:** Participant demographic data (*n* = 1,092).

**Demographic variable**	**Mean ±SD or *n* (%)**
Age	20.05 ± 1.72
**Gender**	
Female	432 (39.6)
Male	660 (60.4)
**Province**	
Occidental region	69 (6.4)
Pinar del Río	1 (0.1)
Artemisa	4 (0.4)
La Habana	61 (5.6)
Isla de la Juventud	1 (0.1)
Mayabeque	1 (0.1)
Matanzas	1 (0.1)
Central Region	1,016 (93.0)
Cienfuegos	44 (4.0)
Villa Clara	839 (76.8)
Sancti Spíritus	38 (3.5)
Ciego de Ávila	23 (2.1)
Camagüey	72 (6.6)
Oriental Region	7 (0.7)
Las Tunas	4 (0.4)
Granma	2 (0.2)
Santiago de Cuba	1 (0.1)

SE, Standard Deviation.

The scale demonstrated adequate psychometric properties across its seven dimensions (Table 2). Mean scores (M) ranged from 3.30 (behavioral regulation) to 4.53 (social behavior), with standard deviations (SD) indicating moderate variability (0.84–1.90). Acceptable item-test correlations (r) were observed (0.35–0.63), particularly for attention (UEF22: r = 0.63) and emotional regulation items (UEF29: r = 0.53), along with optimal factor loadings (>0.70) for responsibility monitoring (UEF9: 0.79), attentional system (UEF22: 0.81), and learning verification (UEF20: 0.81). However, the behavioral regulation dimension showed the lowest loadings (UEF19: 0.46; UEF18: 0.49), suggesting weaker internal consistency.

**Table 2:** Descriptive statistics, item-test correlations (*r*), and loadings of the UEF-1 items.

**Items**	***M***	***SD***	***r***	**Loadings**
***F1 Conscious monitoring of responsibilities***				
UEF2 Puedo terminar una tarea universitaria cuando es muy larga//*I can finish a university assignment when it is very long*.	4.01	1.04	0.50	0.70
UEF8 Puedo realizar las tareas universitarias de forma independiente y sin ayuda de los demás/*I can complete college assignments independently and without help from others*.	3.80	1.90	0.43	0.63
UEF9 Logro realizar exitosamente mis trabajos de la universidad/*I successfully complete my university assignments*.	4.17	0.89	0.55	0.79
UEF15 Puedo realizar mis trabajos sin que alguien me supervise/*I can do my assignments without someone supervising me*.	4.14	0.99	0.41	0.61
UEF27 Termino mis tareas universitarias a tiempo/*I finish my college assignments on time*.	4.07	1.03	0.54	0.77
***F2 Supervisory Attention System***				
UEF10 Tengo buena concentración/*I can concentrate well*.	3.60	1.21	0.60	0.77
UEF14 Soy capaz de mantener la atención en una actividad/*I can maintain my attention on an activity*.	3.96	1.07	0.59	0.74
UEF22 Me es fácil concentrarme en mis actividades universitarias/*It is easy for me to concentrate on my college activities*.	3.70	1.11	0.63	0.81
UEF28 Mantengo buenos hábitos de estudio/*I maintain good study habits*.	3.47	1.26	0.57	0.73
UEF13 Me concentro en mis actividades universitarias, dejando de lado las cosas irrelevantes/*I focus on my university activities, leaving irrelevant things aside*.	3.35	1.21	0.59	0.74
***F3 Conscious regulation of behavior***				
UEF3 Actúo siempre pensando y reflexionando las consecuencias de mis actos/*I always act thinking and reflecting on the consequences of my actions*.	3.87	1.17	0.46	0.56
UEF11 Puedo estar quieto/a y tranquilo/a mientras espero/*I can be still and calm while I wait*.	3.30	1.43	0.42	0.54
UEF16 Me es fácil comportarme adecuadamente en las reuniones sociales/*It is easy for me to behave appropriately in social gatherings*.	4.53	0.84	0.47	0.66
UEF17 Cuando alguien me lo pide, puedo dejar con facilidad de hacer algo que me distrae/*When someone asks me to, I can easily stop doing something that distracts me*.	4.01	1.07	0.47	0.59
UEF18 Dejo hablar a los demás, sin hacer interrupciones/*I let others speak, without interrupting*.	4.13	1.05	0.38	0.49
UEF19 Puedo anticipar las consecuencias de mis actos	3.95	1.04	0.35	0.46
***F4 Verification of behavior to learn***				
UEF20 Verifico que mis tareas universitarias estén bien realizadas y sin errores, antes de presentarlas al profesor/*I verify that my university assignments are well done and without errors, before giving them to the professor*.	4.12	1.08	0.52	0.81
UEF23 Reviso la ortografía y redacción de mis tareas universitarias antes de finalizarlas/*I check the spelling and wording of my college assignments before I finish them*.	4.17	1.23	0.39	0.67
UEF24/*I remember to take home assignments, materials, or college papers*.	4.06	1.22	0.46	0.71
UEF30 Al finalizar una actividad universitaria, verifico que haya logrado lo planificado*/At the end of a university activity, I verify that I have achieved what I planned*.	4.06	1.06	0.56	0.81
***F5 Decision making***				
UEF5 Tengo la capacidad para tomar decisiones en forma independiente/*I can make decisions independently*.	4.29	0.95	0.43	0.64
UEF12 Tengo la capacidad para resolver problemas en la universidad como en mi vida personal/*I can solve problems at university as well as in my personal life*.	4.12	0.96	0.55	0.80
UEF21 Puedo tomar decisiones sin dificultad, incluso ante las cosas más complicadas/*I can make decisions without difficulty, even in the most complicated things*.	3.85	1.07	0.51	0.73
***F6 Conscious regulation of emotions***				
UEF4 Regulo adecuadamente mis emociones/*I properly regulate my emotions*.	3.65	1.17	0.49	0.77
UEF25 Mantengo la calma con facilidad/*I can keep calm easily*.	3.65	1.20	0.45	0.69
UEF29 Tengo un estado de ánimo estable/*My moods are stable*.	3.66	1.29	0.53	0.82
UEF31 Soy capaz de regular mis emociones/*I can regulate my emotions*.	3.78	1.20	0.50	0.84
***F7 Management of elements to solve tasks***				
UEF1 Tengo facilidad para recoger y dejar ordenadas mis cosas cuando se me pide que lo haga/*It is easy to collect and leave my things organized when asked to do so*.	4.01	1.11	0.50	0.75
UEF6 Tengo mis cosas en el lugar adecuado y en orden/*I have my things in the right place and organized*.	3.65	1.31	0.51	0.78
UEF7 Tengo facilidad para encontrar rápidamente mis materiales al buscarlos en mi cuarto o escritorio/*I have an easy time finding my materials by looking for them in my room or desk*.	3.94	1.25	0.46	0.71
UEF26 Recojo mi desorden sin que otros lo hagan por mí/*I pick up my mess without others having to do it for me*	4.13	1.64	0.41	0.66

*M*, mean; *SD*, standard deviation; *r*, correlation index.

## Limitations

Among the study's limitations is the geographic bias in the sample, which was predominantly composed of universities from the Central Region of Cuba (93.0%), with limited representation from the Western (6.4%) and Eastern (0.7%) regions. This imbalance may affect the generalizability of the results, as the socio-educational particularities of other regions—such as access to resources, institutional dynamics, or student profiles—are not adequately reflected.

Additionally, the sample's representativeness may be constrained, as it focuses exclusively on universities in Cuba, potentially missing the diversity of university students across Latin America and other regions. The reliance on self-reported data from the Executive Function Scale (UEF-1) may introduce biases, such as social desirability or inaccuracies in self-assessment, which could impact the validity of the results.

Moreover, the study's specific context—conducted within the socio-cultural and educational environment of Cuba—may limit the generalizability of the findings. Differences in educational systems, socio-economic conditions, and cultural norms may affect executive function performance and perceptions differently in other contexts. Finally, external factors, such as peer influence and the presence of instructors, could influence participants' responses and affect the overall data quality.

## Data Availability

The datasets presented in this study can be found in online repositories. The names of the repository/repositories and accession number(s) can be found in the article/supplementary material.
